# Strain-Dependent Host Transcriptional Responses to *Toxoplasma* Infection Are Largely Conserved in Mammalian and Avian Hosts

**DOI:** 10.1371/journal.pone.0026369

**Published:** 2011-10-13

**Authors:** Yi-Ching Ong, Jon P. Boyle, John C. Boothroyd

**Affiliations:** 1 Stanford University, Department of Microbiology and Immunology, Stanford, California, United States of America; 2 University of Pittsburgh, Department of Molecular Biology, Pittsburgh, Pennsylvania, United States of America; French National Centre for Scientific Research, France

## Abstract

Toxoplasma gondii has a remarkable ability to infect an enormous variety of mammalian and avian species. Given this, it is surprising that three strains (Types I/II/III) account for the majority of isolates from Europe/North America. The selective pressures that have driven the emergence of these particular strains, however, remain enigmatic. We hypothesized that strain selection might be partially driven by adaptation of strains for mammalian versus avian hosts. To test this, we examine in vitro, strain-dependent host responses in fibroblasts of a representative avian host, the chicken (Gallus gallus). Using gene expression profiling of infected chicken embryonic fibroblasts and pathway analysis to assess host response, we show here that chicken cells respond with distinct transcriptional profiles upon infection with Type II versus III strains that are reminiscent of profiles observed in mammalian cells. To identify the parasite drivers of these differences, chicken fibroblasts were infected with individual F1 progeny of a Type II x III cross and host gene expression was assessed for each by microarray. QTL mapping of transcriptional differences suggested, and deletion strains confirmed, that, as in mammalian cells, the polymorphic rhoptry kinase ROP16 is the major driver of strain-specific responses. We originally hypothesized that comparing avian versus mammalian host response might reveal an inversion in parasite strain-dependent phenotypes; specifically, for polymorphic effectors like ROP16, we hypothesized that the allele with most activity in mammalian cells might be less active in avian cells. Instead, we found that activity of ROP16 alleles appears to be conserved across host species; moreover, additional parasite loci that were previously mapped for strain-specific effects on mammalian response showed similar strain-specific effects in chicken cells. These results indicate that if different hosts select for different parasite genotypes, the selection operates downstream of the signaling occurring during the beginning of the host's immune response.

## Introduction

The Apicomplexan parasite *Toxoplasma gondii* is unique among known eukaryotic pathogens in its extraordinarily broad host range. *In vitro,* it can infect virtually any eukaryotic cell; although its only known definitive hosts are felines, *Toxoplasma'*s known intermediate hosts include a wide range of warm-blooded vertebrates around the world, from avians to mammals. As these parasites are transmitted via carnivorism and ingestion of tissue cysts from infected animals, these intermediate hosts represent an important part of *Toxoplasma's* life cycle.

To infect a given host productively, *Toxoplasma* must be able to achieve the right balance with the host's immune response. If the response to infection is too weak and parasites are allowed to proliferate unchecked, the infection will overwhelm the host; but if the immune response to infection is too robust, it can lead to host immunopathology. The importance of this balance has been underscored by several studies examining the role of various host immune factors in resistance to infection. In mice deficient in key pro-inflammatory effectors such as IL-12, IFN-γ, and p47 GTPases, even mouse-avirulent strains of *Toxoplasma* cause a lethal infection characterized by extremely high parasite burden and widespread dissemination [1]. In the converse scenario, where mice are deficient in key regulatory cytokines such as IL-10 and IL-27, immunopathology resulting from infection is increased [2], [3].

*Toxoplasma* has evolved a battery of strategies for the purpose of modulating host immunity as reviewed elsewhere [4], [5]. Interestingly, the success of these strategies in murine hosts appears to vary dramatically by parasite strain. Strain-dependence has been observed for a range of phenotypes including blockade of host apoptosis [6], evasion of p47 GTPase-mediated killing [7], production of IL-12 [8], intersection of MAPK signaling [9], induction of NF-κB signaling [10], and induction and sustenance of JAK/STAT signaling [11]. It is therefore not surprising that where specific parasite effectors underlying these phenotypes have been identified and characterized, they are either highly polymorphic between different parasite strains [11], [12], [13] and/or differentially expressed between strains [9], [14], [15].

Diversity among *Toxoplasma* strains is expected given the diversity among its hosts. Strikingly, however, just three clonal lineages – Type I, II, and III – account for the majority of clinical and natural isolates in Europe and North America [16]. Moreover, genotypic analysis of these strains, as well as some of the so-called ‘atypical’ strains that do not belong to one of these three lineages (including a recently described Type IV/X lineage found in North American animals [17], [18]), has demonstrated that there are generally only two major allelic types for each locus [19]. Consistent with this, a genealogy of the three major strains suggests that the Type I and III strains are each descendants of a cross between a Type II strain and one of two closely related ancestral strains [20].

This raises the question of what kind of selective pressures might have culminated in the emergence of especially the Types I and III strains and driven selection for their particular polymorphic alleles. For example, the Type I and III alleles encoding the secreted rhoptry kinase ROP16 are suggested to play an important role in dampening host inflammation by activation of human and murine STAT3 and STAT6, an activity that may yield important benefits for the parasite [21], [22]. Yet the Type II allele has been maintained and selected for, despite its apparently decreased activity vis-à-vis the STATs in human and murine cells resulting from a single amino acid difference in the kinase domain [23]. Polymorphisms in key parasite effectors likely reflect parasite adaptation for host niches of particular importance in transmission [24]; however, the nature of these host niches remains mysterious. One hypothesis is that avian and mammalian hosts, being evolutionarily divergent from each other, might represent two distinct and important host niches and thereby partially explain the allelic dimorphism observed in *Toxoplasma* strains. To return to the example of ROP16, this hypothesis predicts that the polymorphism that renders the Type II allele less active towards murine and human STATs might actually promote its affinity for and activity towards avian STATs, leading to a host-dependent inversion of the observed phenotypic differences between strains.

*Toxoplasma* infects a broad range of avian species, from passeriform birds like sparrows to domesticated birds like chickens [25]. Very little is known, however, about the avian host response to *Toxoplasma* infection, particularly how it varies by parasite genotype. Some clues, however, come from studies of chickens which may represent an important source of infection for humans due to their ground-feeding habits that make them highly likely to ingest parasite oocysts [26]. Generally, chickens are considered to be refractory to severe *Toxoplasma* infection*;* in studies where chickens were inoculated with high oocyst loads of either Type I or Type II parasites, infection was confirmed by isolation of tissue cysts, but no symptoms of disease were detected [27], [28]. This is strikingly different from the pattern observed in mice, where Type I parasites have an LD_100_ of just one parasite. Outside of experimental infections, neurological signs of toxoplasmosis have been observed in chickens only in rare instances; the strains isolated from these chickens were preliminarily genotyped as Type II [29]. It is possible, however, that these strains might actually be less common ‘atypical’ strains, as the few loci that were used for the genotyping have been shown to yield less than complete information [20].

We therefore set out to investigate whether and how avian host response to *Toxoplasma* infection varies by parasite strain. We chose to use chickens as a representative avian host for this study as they are highly infected in nature [26]. Moreover, because of the chicken's importance to agriculture and as a model for vertebrate development, a richer toolkit has been developed for their study than is available for other avian species. Using transcriptomic profiling of chicken embryonic fibroblasts and pathway analysis to assess host response, we show here that chicken cells do indeed respond with distinct host transcriptional profiles upon infection with different strains. QTL analysis of these transcriptional differences was used to map the parasite loci involved and the results compared with previous studies in human fibroblasts. The implications of the results for the evolution of *Toxoplasma* strain differences are discussed.

## Materials and Methods

### Host cell culture and parasites

SL-29 primary chicken embryonic fibroblasts (CRL-1590; ATCC, Manassas, VA) were maintained in Dulbecco's modified Eagle's medium (30–2002; ATCC, Manassas, VA) supplemented with 5% FCS (Hyclone, Logan, UT) and 5% tryptose phosphate broth (Sigma, St. Louis, MO). Primary chicken embryonic fibroblasts (CEFs) were derived from specific-pathogen-free fertilized eggs purchased from Charles River (Wilmington, MA). Fibroblasts were prepared from 12-day old embryos as described elsewhere [30], and maintained in complete DMEM, comprised of Dulbecco's modified Eagle's medium (Invitrogen, Carlsbad, CA) supplemented with 10% heat-inactivated fetal calf serum (FCS; Hyclone, Logan, UT), 2 mM L-glutamine, 100 U ml^−1^ penicillin and 100 µg ml^−1^ streptomycin. For CEF maintenance, complete DMEM was additionally supplemented with 1% heat-inactivated chicken serum (Invitrogen, Carlsbad, CA), and 1 mM sodium pyruvate (Invitrogen, Carlsbad, CA).

HFF (human foreskin fibroblasts [31]) were maintained in complete DMEM as described previously [31]. *Toxoplasma gondii* tachyzoites were maintained *in vitro* by serial passage on confluent monolayers of HFF in complete DMEM at 37°C with 5% CO_2_, as previously described [32]. The Type I RH and mutant RH*Δrop16* (“ROP16-KO”) strains have been described elsewhere [33]; the Type II (ME49), Type III (CEP), and IIxIII F1 progeny have also been described elsewhere [11]. F1 progeny used for this study were: C96A5, C96B4, C96C12, C96E7, C96H6, STD3, STF3, STG4, STC7, STC8, STD10, STE10, S2T, CL13, CL16, CL29, S21, S23, S27, S28, and S30. Parasites were tested for mycoplasma contamination at regular intervals and contamination was not detected.

### Microarray analysis

Parasites were harvested by syringe-lysis and washed twice in 35 ml complete DMEM, followed by filtration through a 5 µm filter (Millipore, Billerica, MA) to remove cell debris. Confluent monolayers of SL-29 or primary CEFs in 6-well plates were infected at MOI ∼3 and total RNA was extracted at the indicated timepoint (5 or 24 hours post-infection) using Trizol (Invitrogen, Carlsbad, CA). Total RNA from each sample was labeled using either the Affymetrix One Cycle Labeling Kit or the Affymetrix 3′ IVT Express Kit as indicated (Affymetrix, Santa Clara, CA). 20 µg of resulting cRNA from each sample was hybridized onto Affymetrix Chicken Genome Array chips. The microarray data is MIAME compliant and the raw data has been deposited in the NCBI Gene Expression Omnibus (GSE29565 http://www.ncbi.nlm.nih.gov/geo/). Gene expression values were computed by implementing the Robust Multichip Average procedure for normalization [34]. Data were subjected to a two-class comparison by Significance Analysis of Microarrays (SAM 2.0) analysis [35] as implemented in MeV v. 4.6.1 from the TM4 software suite [36]. Genes meeting the threshold of a<5% false-discovery rate (FDR) and absolute expression fold change greater than 1.5 were considered as significantly differentially expressed. For the genome-wide scan, R/QTL analysis was performed as described previously [21], [37]. To determine significance, p-values were calculated based on 500 permutations; genes that mapped to a parasite genetic locus with a p-value of <0.05 were considered significant.

### Pathway analysis and functional annotation

Gene set enrichment analysis (GSEA) was used to find candidate transcription factors and canonical pathways that were activated or induced upon infection [38], [39]. This program makes use of defined gene sets that were generated experimentally, computationally, or by curation of literature. It then allows for comparison of ranked lists of genes to these reference sets and determines whether members of these reference sets are randomly distributed throughout the ranked lists (suggesting no overlap in the biology of these sets) or primarily found at the top or bottom of that list (suggesting enrichment). For the purposes of hypothesis-generation, gene sets enriched with a false discovery rate (FDR) <0.25 were considered significant. The following gene sets from the Molecular Signatures Database were evaluated for enrichment: c2.cgp.v3.0 (gene sets derived from literature where cells were subjected to either chemical or genetic perturbations), c2.kegg (gene sets derived from KEGG canonical pathway lists), and c3.tft.v3.0 (gene sets predicted on the basis of a common cis-regulatory motif conserved in the human, mouse, rat, and dog genomes) [40]. Identification of functionally-related gene groups enriched in gene sets of interest was performed using DAVID 6.7, available at http://david.abcc.ncifcrf.gov/ [41], [42].

## Results

### Type II strains and Type III strains induce distinct transcriptional profiles in infected chicken embryonic fibroblasts

**Figure 1:**
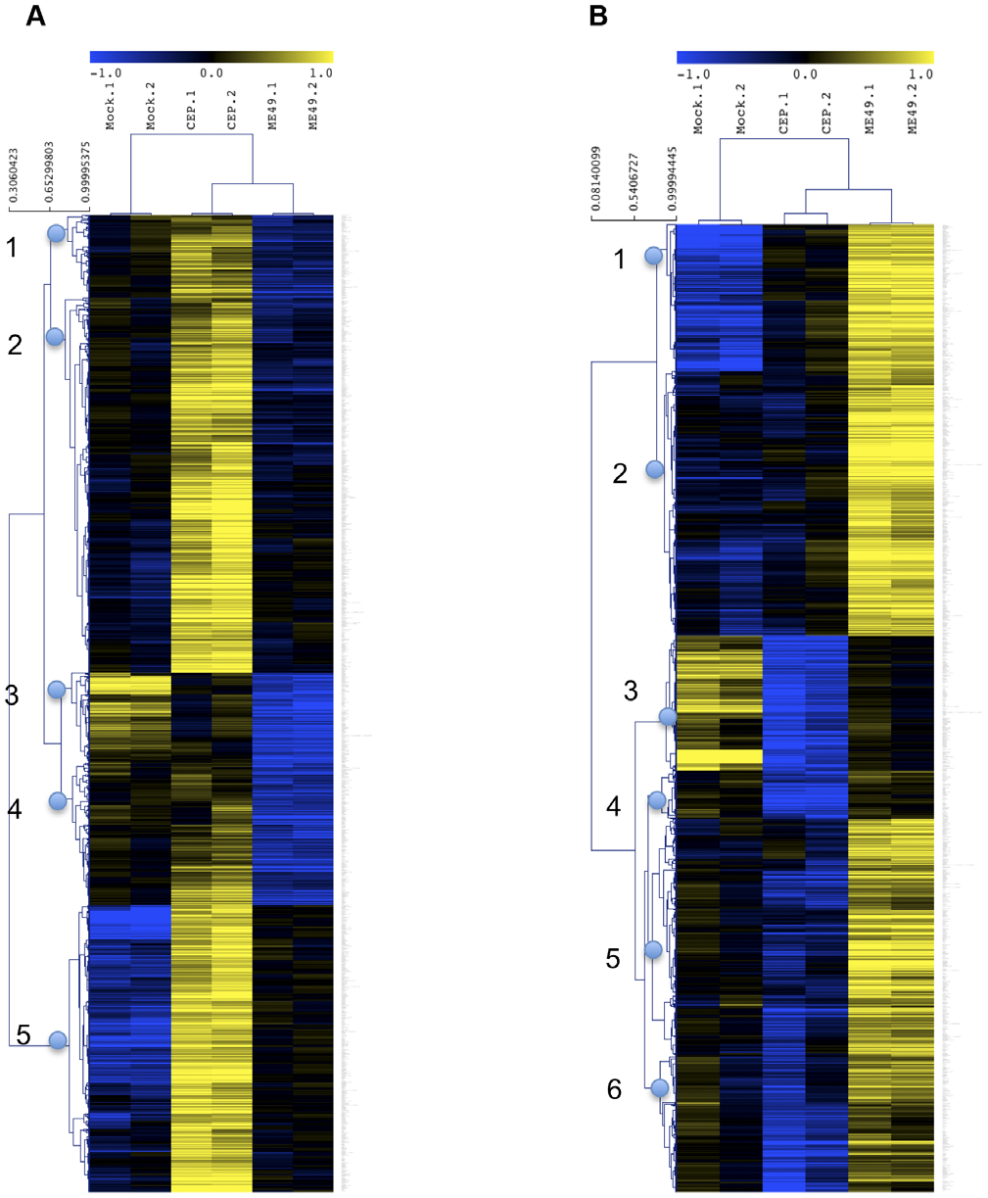
Type II and Type III strains induce distinct transcriptional programs in chicken embryonic fibroblasts. Microarray analysis of SL-29 chicken embryonic fibroblasts subjected to mock, Type II (ME49), or Type III (CEP) infection. 2 biological replicates were performed for each treatment and RNA was harvested at 24 hours post-infection. Genes shown are those identified as significantly up-regulated by SAM analysis (FDR<5% and fold change ≥1.5) either in Type III infection over Type II infection (*A*) or in Type II infection over Type III infection (*B*). For heatmap representation, probe intensities were log2-transformed and median centered by row; unsupervised hierarchical clustering was then performed (cluster numbers indicated on left). The color gradient key at top represents the color range in a log2 scale, from relative upregulation by 2-fold (yellow) and relative downregulation by 2-fold (blue).

Strain-dependent host response to the widespread *Toxoplasma* strains Type II and Type III has been extensively characterized at the transcriptional level in human foreskin fibroblasts [11], [22] and murine macrophages [43]. To test the hypothesis that these strains might elicit different host responses in avian cells, we infected SL-29 primary chicken embryonic fibroblasts (SL-29s) with Type II and Type III parasites and analyzed host gene expression 24 hours post-infection by Affymetrix microarrays. Significance Analysis of Microarrays (SAM) identified 432 genes that were significantly up-regulated (≥1.5-fold-change and FDR<5%) in cells infected with Type III versus Type II strains, and 450 genes that were significantly up-regulated by the same criteria in Type II versus Type III infections (Figure 1A and B). In some cases, both strains induced up-regulation of gene expression relative to mock-infected cells (cluster 5 in Fig 1A, cluster 1 in Fig. 1B), but the degree of up-regulation differed between strains. In other cases, the directionality of the change in gene expression relative to mock-infected cells was inverted depending on the strain (cluster 4 in Fig. 1A, clusters 5 and 6 in 1B).

To investigate which signaling pathways might be implicated in these strain-specific differences in expression profiles, we used Gene Set Enrichment Analysis (GSEA), which identifies gene sets that previous microarray experiments have reported to be coordinately regulated by any of a large number of conditions. Analysis of genes that were more highly expressed in the Type III-infected cells vs. Type II infections revealed significant overlap with gene sets associated with cell proliferation or oncogenic transformation (Table 1), whereas analysis of genes more highly expressed in Type II-infections identified gene sets up-regulated in response to TNF-signaling, interferon-stimulation, and NF-κB-stimulation (Table 2). Consistent with this, analysis using DAVID software to identify enriched biological themes and functional annotations showed that genes with higher expression in Type III-infected cells vs. Type II-infections were enriched in functional annotations for cellular adhesion, motility, proliferation, and JAK/STAT signaling (Table 3), whereas genes with higher expression in Type II-infected cells were enriched in functional associations with leukocyte/lymphocyte regulation and apoptosis (Table 4).

**Table 1:** Gene sets from the Molecular Signatures Database CGP (chemical and genetic perturbations) library identified by GSEA as significantly enriched in Type III-induced genes.

NAME	FDR q-val
NIKOLSKY_BREAST_CANCER_17Q11_Q21_AMPLICON	0.04
SAKAI_CHRONIC_HEPATITIS_VS_LIVER_CANCER_DN	0.05
RORIE_TARGETS_OF_EWSR1_FLI1_FUSION_DN	0.05
SENGUPTA_NASOPHARYNGEAL_CARCINOMA_WITH_LMP1_DN	0.06
BEIER_GLIOMA_STEM_CELL_UP	0.09
GAUSSMANN_MLL_AF4_FUSION_TARGETS_D_UP	0.10
SATO_SILENCED_EPIGENETICALLY_IN_PANCREATIC_CANCER	0.10
RICKMAN_HEAD_AND_NECK_CANCER_F	0.10
WANG_BARRETTS_ESOPHAGUS_UP	0.11
KLEIN_PRIMARY_EFFUSION_LYMPHOMA_UP	0.15
GU_PDEF_TARGETS_DN	0.15
NAKAYAMA_SOFT_TISSUE_TUMORS_PCA2_DN	0.15
ROYLANCE_BREAST_CANCER_16Q_COPY_NUMBER_UP	0.16
SMID_BREAST_CANCER_RELAPSE_IN_BONE_UP	0.17
LI_CISPLATIN_RESISTANCE_DN	0.21
RICKMAN_HEAD_AND_NECK_CANCER_E	0.22
HELLER_SILENCED_BY_METHYLATION_DN	0.22
HANN_RESISTANCE_TO_BCL2_INHIBITOR_DN	0.23
BROWNE_HCMV_INFECTION_18HR_DN	0.23
VALK_AML_WITH_FLT3_ITD	0.23

Gene sets with a false-discovery rate (FDR) q-val≤0.25 were considered significant and are listed here.

**Table 2:** Gene sets from the Molecular Signatures Database CGP (chemical and genetic perturbations) library identified by GSEA as significantly enriched in Type II-induced genes.

NAME	FDR q-val
SANA_TNF_SIGNALING_UP	0
HESS_TARGETS_OF_HOXA9_AND_MEIS1_DN	0.04
JAZAERI_BREAST_CANCER_BRCA1_VS_BRCA2_DN	0.04
HINATA_NFKB_TARGETS_KERATINOCYTE_UP	0.04
FARMER_BREAST_CANCER_CLUSTER_1	0.05
LINDSTEDT_DENDRITIC_CELL_MATURATION_A	0.05
HINATA_NFKB_TARGETS_FIBROBLAST_UP	0.07
DAZARD_RESPONSE_TO_UV_SCC_UP	0.07
TRAYNOR_RETT_SYNDROM_UP	0.08
REN_ALVEOLAR_RHABDOMYOSARCOMA_UP	0.08
BROWNE_INTERFERON_RESPONSIVE_GENES	0.08
CHEN_NEUROBLASTOMA_COPY_NUMBER_GAINS	0.09
DER_IFN_GAMMA_RESPONSE_UP	0.09
DUTTA_APOPTOSIS_VIA_NFKB	0.13
XU_HGF_SIGNALING_NOT_VIA_AKT1_48HR_UP	0.13
ZUCCHI_METASTASIS_DN	0.13
TARTE_PLASMA_CELL_VS_B_LYMPHOCYTE_DN	0.14
XU_HGF_TARGETS_REPRESSED_BY_AKT1_DN	0.14
GEISS_RESPONSE_TO_DSRNA_UP	0.15
ZHANG_RESPONSE_TO_IKK_INHIBITOR_AND_TNF_UP	0.15
COLIN_PILOCYTIC_ASTROCYTOMA_VS_GLIOBLASTOMA_DN	0.15
LIANG_SILENCED_BY_METHYLATION_2	0.16
LINDSTEDT_DENDRITIC_CELL_MATURATION_B	0.25

Gene sets with a false-discovery rate (FDR) q-val≤0.25 were considered significant and are listed here.

**Table 3:** Functional categories identified by DAVID as significantly enriched (FDR<25%) in Type III-induced genes.

Term	FDR (%)
GO:0005576∼extracellular region	0.0000033
gga04512:ECM-receptor interaction	0.0022
signal	0.0077
GO:0044421∼extracellular region part	0.0089
signal peptide	0.042
gga04510:Focal adhesion	0.062
GO:0031012∼extracellular matrix	0.18
glycoprotein	0.18
GO:0040008∼regulation of growth	0.43
Secreted	0.38
GO:0009968∼negative regulation of signal transduction	0.52
GO:0005578∼proteinaceous extracellular matrix	0.50
GO:0010648∼negative regulation of cell communication	0.70
GO:0045177∼apical part of cell	0.56
GO:0007167∼enzyme linked receptor protein signaling pathway	0.96
GO:0007155∼cell adhesion	1.08
GO:0022610∼biological adhesion	1.08
disulfide bond	0.96
IPR000980:SH2 motif	1.25
GO:0005887∼integral to plasma membrane	1.11
glycosylation site:N-linked (GlcNAc…)	1.41
GO:0031226∼intrinsic to plasma membrane	1.28
IPR001496:SOCS protein, C-terminal	1.94
SM00252:SH2	2.15
GO:0051240∼positive regulation of multicellular organismal process	3.28
SM00253:SOCS	2.58
GO:0006928∼cell motion	4.63
disulfide bond	6.54
GO:0007169∼transmembrane receptor protein tyrosine kinase signaling pathway	8.40
GO:0001568∼blood vessel development	8.84
GO:0007242∼intracellular signaling cascade	10.62
GO:0001944∼vasculature development	10.75
Signal transduction inhibitor	8.49
IPR013320:Concanavalin A-like lectin/glucanase, subgroup	9.92
GO:0031175∼neuron projection development	11.93
GO:0051094∼positive regulation of developmental process	12.34
GO:0030030∼cell projection organization	13.48
Immunoglobulin domain	10.33
GO:0040014∼regulation of multicellular organism growth	15.59
GO:0001525∼angiogenesis	16.06
gga04630:Jak-STAT signaling pathway	10.60
GO:0048514∼blood vessel morphogenesis	16.36
short sequence motif:Cell attachment site	13.94
IPR013098:Immunoglobulin I-set	14.57
GO:0005886∼plasma membrane	13.72
IPR012680:Laminin G, subdomain 2	15.80
GO:0019838∼growth factor binding	16.18
IPR001791:Laminin G	19.14

**Table 4:** Functional categories identified by DAVID as significantly enriched (FDR<25%) in Type II-induced genes.

Term	FDR (%)
GO:0044421∼extracellular region part	0.0016
GO:0005576∼extracellular region	0.0027
GO:0051249∼regulation of lymphocyte activation	0.016
GO:0002694∼regulation of leukocyte activation	0.025
GO:0050865∼regulation of cell activation	0.044
GO:0042981∼regulation of apoptosis	0.093
GO:0043067∼regulation of programmed cell death	0.11
GO:0010941∼regulation of cell death	0.12
GO:0050863∼regulation of T cell activation	0.12
GO:0005125∼cytokine activity	0.12
GO:0006955∼immune response	0.19
GO:0043068∼positive regulation of programmed cell death	0.19
GO:0043065∼positive regulation of apoptosis	0.19
GO:0010942∼positive regulation of cell death	0.21
GO:0002250∼adaptive immune response	0.21
GO:0002460∼adaptive immune response based on somatic recombination of immune receptors built from immunoglobulin superfamily domains	0.21
GO:0045619∼regulation of lymphocyte differentiation	0.65
GO:0051251∼positive regulation of lymphocyte activation	0.73
GO:0005615∼extracellular space	0.58
GO:0002696∼positive regulation of leukocyte activation	0.93
GO:0002684∼positive regulation of immune system process	0.97
GO:0042127∼regulation of cell proliferation	0.97
GO:0050867∼positive regulation of cell activation	1.17
GO:0019724∼B cell mediated immunity	1.58
GO:0002449∼lymphocyte mediated immunity	2.03
GO:0031012∼extracellular matrix	2.69
GO:0002443∼leukocyte mediated immunity	3.79
cytokine	3.24
GO:0043383∼negative T cell selection	4.50
GO:0045060∼negative thymic T cell selection	4.50
signal peptide	3.69
GO:0008285∼negative regulation of cell proliferation	4.76
GO:0050870∼positive regulation of T cell activation	5.14
GO:0000122∼negative regulation of transcription from RNA polymerase II promoter	5.33
GO:0045580∼regulation of T cell differentiation	5.34
gga04514:Cell adhesion molecules (CAMs)	3.74
GO:0046649∼lymphocyte activation	7.99
GO:0048048∼embryonic eye morphogenesis	9.03
GO:0005578∼proteinaceous extracellular matrix	6.78
GO:0030098∼lymphocyte differentiation	9.60
GO:0042110∼T cell activation	11.20
GO:0045061∼thymic T cell selection	11.73
GO:0051250∼negative regulation of lymphocyte activation	11.73
GO:0002695∼negative regulation of leukocyte activation	11.73
signal	9.10
GO:0045321∼leukocyte activation	13.40
GO:0009986∼cell surface	10.01
GO:0002252∼immune effector process	14.67
Secreted	11.18
GO:0050866∼negative regulation of cell activation	17.84
GO:0045089∼positive regulation of innate immune response	17.84
GO:0007155∼cell adhesion	20.73
GO:0022610∼biological adhesion	20.73
GO:0045088∼regulation of innate immune response	21.17
GO:0016064∼immunoglobulin mediated immune response	21.17
GO:0002521∼leukocyte differentiation	21.27
GO:0001775∼cell activation	22.37
GO:0030217∼T cell differentiation	22.77
GO:0005604∼basement membrane	17.39
GO:0009897∼external side of plasma membrane	17.72
GO:0016564∼transcription repressor activity	20.13
GO:0045892∼negative regulation of transcription, DNA-dependent	24.25
GO:0050778∼positive regulation of immune response	24.56
GO:0045058∼T cell selection	24.62
GO:0045596∼negative regulation of cell differentiation	26.21
gga00982:Drug metabolism	17.83
gga00980:Metabolism of xenobiotics by cytochrome P450	17.83

To further dissect signaling pathways that were perturbed in a strain-specific manner, we attempted to use GSEA to identify transcription factor binding sites (TFBSs) enriched in genes specifically up-regulated by either Type II or Type III infection. The only TFBSs identified as significantly enriched (FDR<0.25) in genes highly expressed during Type II infection were NF-κB (FDR of 0.228) and PBX1 (FDR of 0.226) (Tables S1 and S2). No TFBSs emerged as significantly enriched in genes highly expressed during Type III infection. This lack of predictive ability for TFBSs in an avian genome is not surprising given that GSEA makes use of reference gene sets that are either predicted to be targets of specific transcription factors based on bioinformatic analysis of conserved motifs, or have been identified as transcription factor targets based on experimental data. In both instances, the GSEA TFBS reference gene sets were generated based on data from select mammalian genomes, thus hindering direct comparison to an avian context.

Overall, these results are reminiscent of the patterns observed for Type II and Type III infection of murine macrophages [43] and HFF [22]. As in the latter two cell lines, the gene signature specific to Type II infection in chicken fibroblasts was found to be significantly enriched in TFBSs for NF-κB. While direct comparison of TFBSs enriched in the gene signature specific to Type III infection of chicken cells vs. mammalian cells is not possible for the reasons discussed above, functional annotation of the Type III-specific signature in chicken fibroblasts bears a strong resemblance to the Type III-specific signature in murine macrophages, which is characterized by enrichment in TFBSs of transcription factors involved in hematopoietic cell proliferation, survival, and differentiation (GATA1, E2F and HOXA9) [43].

### Genome-wide scan locates QTLs in the parasite genome corresponding to strain-specific host gene expression

**Figure 2:**
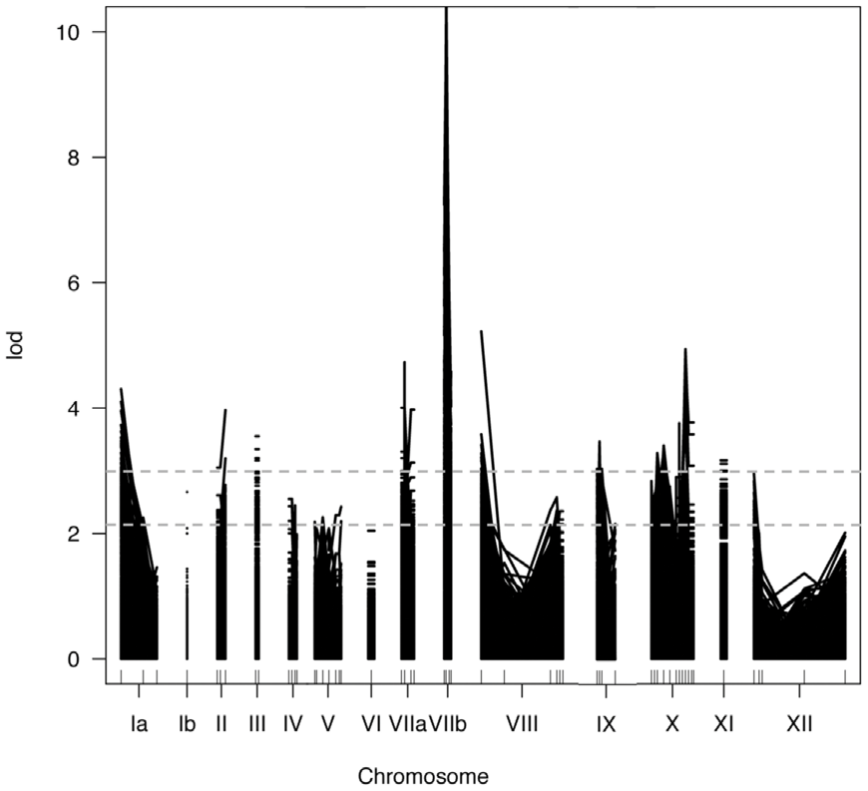
Genome-wide QTL map of strain-specific differences in host gene expression in infected chicken embryonic fibroblasts. SL-29 chicken embryonic fibroblasts were infected with 21 F1 progeny from a Type II x Type III cross and host gene expression at 24 hours post-infection was profiled by microarray. A one-dimensional genome-wide scan was conducted to identify *Toxoplasma* genetic markers associated with the expression level of each of the host genes represented on the microarray. The output of this QTL analysis is graphed here, where each line represents a host gene that mapped with a LOD score >2 to a *Toxoplasma* locus. To determine significance, p-values were calculated based on 500 permutations; genes that mapped to a parasite genetic locus with a p-value of <0.05 were considered significant. Threshold LOD scores for this p-value varied on a gene-by-gene basis from 2.10 to 3.14 and are represented by the two dotted gray lines overlaying the graph.

To further dissect the basis of the strain-specific transcriptional profiles observed above, we set out to identify the parasite loci involved using an approach that has previously revealed several key parasite effectors operating in infection of human fibroblasts [21]. This approach is based on the premise that if a host response phenotype is strain-specifically regulated and has a basis in parasite genotype, it should segregate among F1 progeny derived from a cross between two strains that differ in that phenotype [11]. Accordingly, SL-29 chicken embryonic fibroblasts were infected with 21 F1 progeny from a Type II x Type III cross and host gene expression at 24 hours post-infection was profiled by microarray as before. We then performed a genome-wide scan for association of *Toxoplasma* genetic markers and the expression level of each of the host genes represented on the microarray. Out of 32,773 chicken-specific probes, representing over 28,000 unique chicken genes, 689 had LOD scores that mapped to a specific *Toxoplasma* genomic locus with a significance level of p<0.05 (calculated by permutation test; Fig. 2 and Table 5). Substantial clusters of host genes (50 or more) mapped to chromosomes Ia, VIIa, VIIb, and X. The presence of a peak on chromosome Ia was intriguing as it is unusually monomorphic among the three major strains of *Toxoplasma*; it has been suggested that it may carry an especially important combination of monomorphic alleles that has facilitated the extraordinary global sweep of these three clonal lineages [44]. This high level of conservation may facilitate future identification of the relevant locus, as a search of ToxoDB v6.4 reveals just 24 predicted genes that contain non-synonymous SNPs between the Type II and Type III genomes. Two of these predicted genes, TGME49_094190 (encoding a protein with homology to 3-hydroxyisobutyryl-CoA hydrolase) and TGME49_095380 (encoding a protein with no known function and no apparent homologues outside the Apicomplexa), contain a putative signal-peptide and may be secreted into the host cell, making them especially good candidates for modulating host cell response in a strain-specific manner [45]. On VIIa, the majority of genes mapped to the CS3 marker, which has previously been associated with the polymorphic rhoptry kinase ROP18 [14], [15]. On VIIb, the majority of genes mapped in the vicinity of the L339 and AK104 markers, which have been previously been associated with the polymorphic rhoptry kinase ROP16 [21]. On chromosome X, there were two distinct peaks; one is at the right end and indicates cosegregation with marker AK154 while the other is at the left and is associated with AK66 and SRS4. AK154 has been previously linked to a key polymorphic dense granule protein, GRA15, which directs strain-specific NF-κB activation [22]. There is no known candidate for a polymorphic locus in the region of AK66 and SRS4 that has been shown to impact host gene expression, although this peak falls in the vicinity of a previously-mapped locus for virulence in mice [15].

**Table 5:** Genome-wide QTL mapping of strain-specific host transcriptional response in chicken embryonic fibroblasts.

	Number of genes mapping	
Chromosome	p<0.05	p<0.05 Type II vs. III fold-change ≥1.5
Ia	115	2
Ib	1	0
II	10	0
III	14	4
IV	1	0
V	0	0
VI	0	0
VIIa	304	39
VIIb	137	98
VIII	22	1
IX	0	0
X	70	16
XI	9	0
XII	6	0

Expression values of each gene were treated as phenotypes and a one-dimensional genome scan to detect major QTL associated with these expression values was performed. For each gene, the parasite genomic locus corresponding to the maximum LOD score was determined. p-values were calculated by permutation test and only genes with maximum LOD scores meeting the p<0.05 cutoff were considered significant.

Although the greatest number of host gene expression differences were found to map significantly to a QTL on chromosome VIIa (Table 5), comparison with the data summarized in Fig. 1 revealed that many of these host genes were not originally identified as substantially and significantly different in expression levels between Type II and Type III infections. This might be due to the threshold set for differential expression (fold-change ≥1.5 and p<0.05) or a limitation in statistical power (n = 2 for both strains) in the original comparison that was overcome in the comparison of multiple progeny strains; it is also possible that epistatic interactions may have masked certain effects of the VIIa locus. Of the genes that mapped to a given parasite locus in the analysis of the F1 progeny and that were identified as significantly different between infection with the Type II and Type III strains (fold change ≥1.5), the majority mapped to chromosome VIIb (Table 5). Functional annotation of the genes mapping to VIIb revealed a significant enrichment of genes involved in tyrosine kinase signaling cascades (SH2 motifs), JAK/STAT signaling pathways, and extracellular matrix interactions (Table 6).

**Table 6:** Functional categories identified by DAVID as significantly enriched (FDR<25%) in the set of genes mapping to chromosome VIIb.

Term	Fold Enrichment	FDR (%)
IPR000980:SH2 motif	17.10	0.021
SM00252:SH2	14.28	0.031
IPR001496:SOCS protein, C-terminal	37.05	0.14
SM00253:SOCS	30.95	0.17
Signal transduction inhibitor	40.01	2.21
GO:0009968∼negative regulation of signal transduction	10.18	8.62
GO:0005576∼extracellular region	2.54	9.44
GO:0010648∼negative regulation of cell communication	9.63	9.98
GO:0031175∼neuron projection development	7.70	17.65
GO:0030182∼neuron differentiation	5.15	18.26
GO:0005578∼proteinaceous extracellular matrix	4.63	18.37
GO:0031012∼extracellular matrix	4.38	21.63
domain:SOCS box	84.84	22.75
gga04910:Insulin signaling pathway	5.41	22.90
Secreted	2.94	23.19
gga04630:Jak-STAT signaling pathway	5.26	24.42

### ROP16 is likely the key locus on chromosome VIIb responsible for the differences in Type III vs. Type II infection of CEFs

Because *ROP16* was previously identified as the key QTL on chromosome VIIb responsible for strain-specific host gene expression in HFFs, specifically STAT-dependent gene expression, we asked whether *ROP16* might again be responsible for the strain-dependent differences in CEF gene expression that maps to this chromosome. The Type I and Type III alleles of *ROP16* are nearly identical and have been shown to have similar activity and Type I strains have been engineered that lack ROP16; hence, we could use such a strain to characterize ROP16-dependent effects in chicken cells. Primary chicken embryonic fibroblasts were infected with either wild-type (WT) or Δ*rop16* (ROP16-KO) Type I parasites at MOI ∼5 and RNA was harvested 5 hours post-infection for microarray analysis. This timepoint differs from the 24 hour post-infection timepoint used for analysis of strain-dependent differences, but was chosen to facilitate comparison to previous studies of ROP16-dependent transcriptional effects in human fibroblasts [33] and murine macrophages [43]. We have found this timepoint to be most useful when dissecting the effects of ROP16, since it has such a rapid effect on STAT activation; for other parasite effectors that act by other mechanisms, a later timepoint may be more useful in revealing their effects on host cells, which is why the initial analysis of strain-dependent differences was conducted at a 24 hour timepoint**.** Based on SAM analysis, 43 probesets representing 37 unique host genes were identified as significantly up-regulated (≥1.5 fold-change, p<0.05) in WT versus ROP16-KO parasites (Table 7). This gene set had significant overlap with the genes identified as mapping to Chromosome VIIb and those that were significantly higher in Type III versus Type II infection (Table 7). Differences between the two sets are expected as genes that are differentially regulated in WT vs. ROP16-KO Type I parasites reflect a dependence on ROP16 expression, but not necessarily in an allele-specific manner (e.g., previous studies have shown that the Type I, II and III alleles of *ROP16* all drive the early activation of STAT3/6 signaling; it is the sustained activation of these proteins that differs between the Type I/III and Type II alleles [33], [46]. Differences in the timepoint of analysis, as discussed above, might also be expected to affect levels of gene expression. Other differences between Type I and Type III strains, including minor differences in the Type I vs. III alleles of *ROP16*, might also partially account for the observed differences.

**Table 7:** Comparison of genes significantly up-regulated by Type I versus Type I Δ*rop16* infection or Type III versus Type II infection.

GENE SYMBOL	Fold Change, I>I Δrop16, 5 hpi	Fold Change, III>II, 24 hpi
CCL17	21.2	399.6
CA2	8.2	25.7
---	7.4	
---	4.1	16.2
CALCA	4.1	
LOC424241	4.1	
CXCR4	3.6	
SOCS1	3.4	14.7
CISH	3.2	5.8
BMP2	3.1	
DOK5	2.9	3.2
PPP1R3C	2.6	6.6
SERPINB2	2.6	6.2
EAF2	2.4	3.6
SOCS2	2.3	6.5
UGP2	2.2	3.6
RDH10	2.1	2.8
KRT14	2.1	2.9
NPTX2	2.0	
---	2.0	5.2
---	1.9	
---	1.9	
SOCS3	1.9	3.8
FST	1.9	4.6
CCL4	1.8	
LOC395581	1.7	
LOC422150	1.7	
TNFRSF1B	1.6	
WDFY2	1.6	4.6
PLK2	1.6	
APPL2	1.6	3.2
SGK1	1.6	
---	1.6	
---	1.5	2.8
MEOX2	1.5	3.2
---	1.5	
SEMA3A	1.5	4.6

All genes identified as significantly up-regulated (≥1.5 fold-change, p<0.05) between chicken embryonic fibroblasts infected with Type I versus Type I Δ*rop16* parasites are shown here. Bold-face font indicates genes identified as mapping to chromosome VIIb. Where more than one probe corresponded to a given gene, the highest fold-change difference is shown.

As an early activator of STAT3/6, ROP16 appears to functionally mimic some of the effects of IL-4 signaling. Consistent with this observation, a ROP16-dependent signature reminiscent of IL-4 and JAK/STAT signaling was observed in CEFs. For instance, the most differentially regulated chicken gene in comparisons of infection with WT versus ROP16-KO Type I parasites or Type III versus Type II parasites is *CCL17* (Table 7). CCL17 is typically elicited in response to IL-4 stimulation and is a Th2-attracting cytokine produced by monocytes and that recruits CD4+/CD25+ regulatory T cells [47]. *CXCR4*, another gene that is highly differentially expressed in the comparisons reported in Table 7, is also characteristic of a Th2 environment and is known to be IL-4 responsive [48]. Taken together, these data suggest that the Type I/III allele of ROP16 may have conserved function in chickens relative to mammals and drive a Th2-like transcriptional program similar to that seen in human fibroblasts and murine macrophages.

## Discussion

We report here that, contrary to our original prediction, strain-dependent differences in the response of CEFs to infection by Type II and Type III strains exhibit broadly similar patterns to those previously reported in murine macrophages and human fibroblasts. Also consistent with previous findings, the polymorphic rhoptry kinase ROP16 was identified as a key player, with the enhanced activation of JAK/STAT pathways by the Type I/III allele of ROP16, relative to the Type II allele, appearing to be conserved in chicken cells. It is highly likely that, as in mammalian cells, ROP16 is able to directly phosphorylate and activate STATs in infected chicken cells. Direct confirmation of this awaits the development of reagents suitable for study of chicken STATs. Of note is that while chicken homologues of IL-4 and its cognate receptor IL-4Rα have been identified [49], chickens lack an identifiable homologue of STAT6 [50], which is the primary mediator of IL-4 signaling in mammalian cells. Chickens do possess a homologue of mammalian STAT5, however, and this might mediate IL-4 signaling in chicken cells; IL-4 has been known to signal through STAT5 as well as STAT6 in human and murine cells [51] and STAT5 has been shown to respond to hematopoietic cytokine signaling in chicken cells [52]. We and others have observed that STAT5 activation in infected HFFs and murine macrophages is ROP16-dependent (data not shown; Jeroen Saeij, personal communication), providing a possible molecular mechanism by which ROP16 might be able to effect IL-4-like signaling in chicken cells.

Our results are not inconsistent with the hypothesis that the selection for particular parasite strains has been driven by the particular requirements of some special host (or hosts) that were historically critical to the evolution of *Toxoplasma.* In initiating this study, we looked to ROP16 as a paradigmatic parasite effector. Studies in murine and human cells showed that in one allelic form, it is capable of driving sustained activation of STAT3 and STAT6, with dramatic consequences for host inflammation, whereas in another allelic form, this activity is much reduced (although not entirely ablated [21]). Given the consequences of ROP16's activity towards the STATs, we reasoned that potent activity should be a universally beneficial one as far as parasite survival. We therefore sought to identify a host in which the ‘inactive’ Type II allele might prove to be actually more active towards the STATs than the Type I/III allele. Since relative differences in STAT-activating capability might be predicted to vary with molecular properties such as substrate binding affinity, we hypothesized that a relevant host context in which the substrate (in this case, the STATs) was as molecularly divergent as possible from murine/human substrates might represent the different niche we hypothesized. Toxoplasma is known to naturally infect only warm-blooded animals, i.e., mammals and birds. Hence, in terms of a molecularly divergent, intracellular context that might reasonably have been a factor in the evolution of Toxoplasma (i.e., in a host with significant transmission capability), avian cells represent an outer limit of difference compared to the exclusively mammalian systems previously studied. Chicken STATs have significant evolutionary distance from mammalian STATs and share only ∼90% amino acid identity with murine STATs [53], [54], leaving open the possibility that their binding interaction with ROP16 might differ in a potentially meaningful way. Our results, however, showed that Type III strains still induce a JAK/STAT enriched gene signature in CEF cells, indicating that the Type I/III allele of ROP16 is still more active than the Type II allele in this different host phylum.

Similarities to strain-specificity between chicken and murine responses were also observed for other Toxoplasma QTLs. For example, we observed a significant difference in the ability of Type II vs. Type III strains to elicit a pro-inflammatory signature enriched in NF-κB-regulated genes, just as has been reported for infection of mammalian cells [22], [43]. NF-κB is conserved in chickens, with ∼70% identity to mammalian NF-κB [55]. Recently, Rosowski and colleagues identified the secreted polymorphic effector GRA15, located on chromosome X, that drives Type II-induced NF-κB activation in murine and human cells and accounts for this characteristic Type II-infection signature [22]. Consistent with this, many genes that were highly expressed in Type II vs. Type III infection of chicken fibroblasts and are known to be typically induced by NF-κB (e.g. *CD83, HHIP*, and *CCL4*) were found to map to chromosome X. Although we have not proven that this difference is in fact due to GRA15, this seems highly likely and suggests that for at least two major, polymorphic effectors, ROP16 and GRA15, and in at least the cell types so far examined, allele-dependent function is conserved in both mammals and avians, despite significant differences in the substrates with which they interact. These findings are consistent with other reports showing that virulence factors may play conserved roles across species and indeed, across kingdoms; this has been strikingly demonstrated for *Pseudomonas aeruginosa* virulence, which relies on the same genetic determinants in an Arabidopsis leaf infiltration model and a mouse full-thickness skin burn model [56].

It remains possible that we have simply not identified the relevant host species of interest and that, in that host species, we would indeed observe an inversion of ROP16's strain-specific phenotype vis-à-vis the STATs and NF-κB. The available evidence, however, is consistent with an alternative model of strain selection wherein the Type II allele of ROP16 is weaker with regard to STAT activation, across all host species. In this scenario, the selection for the Type II allele would come from the fact that inducing such strong and sustained activation of STATs might not be beneficial to the parasite in all hosts. As previously, STAT activation by ROP16 is associated with a driving of infected cells towards a Th2 response [21], [43]. It might be that in a host already predisposed towards a Th2 response, additional suppression of inflammation may prove deleterious for the parasite. It is known, for instance, that some laboratory strains of mice (e.g. Balb/C) are Th2-inclined compared to others (e.g., C57Bl/6), in part due to variations in MHC receptors and cytokine production [57]; it might be the case that some important evolutionary host of *Toxoplasma* in the wild, perhaps some rodent or avian species, is already Th2-inclined. Hence, it might be to the parasite's advantage (i.e., transmission would be enhanced) if the infecting strain of *Toxoplasma* did not further dampen inflammation by strongly activating STAT3/6. Alternatively, the nature of other infections that might co-exist with *Toxoplasma* in a given host species might have demanded (i.e., selected) for a *Toxoplasma* strain that pushes the immune response in a Th1 or Th2 direction. For example, worm infections are associated with Th2 responses and so whether a host species is generally infected with worms might represent a significant variable in the optimal interaction of *Toxoplasma* with that host.

The virulence and success of a pathogen such as *Toxoplasma* is determined by both host and parasite factors. As such, a full understanding of its pathogenesis and population biology must take into account the possible interactions between these variables. *Toxoplasma* affords a rich system for further exploration in this vein as we learn more about polymorphic effectors such as ROP16 (the parasite ‘variables’) and how these effectors modulate the outcome of infection across the many different host contexts the parasite encounters.

## Supporting Information

Table S1 **Genes identified by GSEA as contributing significantly to the assessment of enrichment for the NF-κB binding motif in Type II-induced genes.** Gene set enrichment analysis (GSEA) was used to find candidate transcription factors induced upon infection; the reference gene set used was c3.tft.v3.0 from the Molecular Signatures Database (comprised of gene sets predicted on the basis of a common cis-regulatory motif conserved in the human, mouse, rat, and dog genomes). An NF-κB motif was identified as significantly enriched (at the FDR<0.25 level) in genes highly expressed during Type II infection. The subset of genes identified as contributing most significantly to this enrichment are listed in the table by gene symbol, along with their rank in gene list (i.e. the position of the gene in the ranked list of genes), rank metric score (correlated to the fold change of gene expression in Type III vs. Type II infection), and running ES (enrichment score). The running ES here indicates the degree to which the reference gene set is overrepresented at the top or bottom of the ranked list of genes differentially expressed in Type II versus III infection. A negative ES indicates gene set enrichment at the bottom of the list, i.e. among genes more highly expressed during Type II infection.
(DOCX)

Table S2 **Genes identified by GSEA as contributing significantly to the assessment of enrichment for the PBX1 binding motif in Type II-induced genes.** Gene set enrichment analysis (GSEA) was used to find candidate transcription factors induced upon infection; the reference gene set used was c3.tft.v3.0 from the Molecular Signatures Database (comprised of gene sets predicted on the basis of a common cis-regulatory motif conserved in the human, mouse, rat, and dog genomes). A PBX1 motif was identified as significantly enriched (at the FDR<0.25 level) in genes highly expressed during Type II infection. The subset of genes identified as contributing most significantly to this enrichment are listed in the table by gene symbol, along with their rank in gene list (i.e. the position of the gene in the ranked list of genes), rank metric score (correlated to the fold change of gene expression in Type III vs. Type II infection), and running ES (enrichment score). The running ES here indicates the degree to which the reference gene set is overrepresented at the top or bottom of the ranked list of genes differentially expressed in Type II versus III infection. A negative ES indicates gene set enrichment at the bottom of the list, i.e. among genes more highly expressed during Type II infection.
(DOCX)
