# Supplementary material for: Strain-Dependent Host Transcriptional Responses to Toxoplasma Infection Are Largely Conserved in Mammalian and Avian Hosts
Source: PLoS One. 2011 Oct 13;6(10):e26369. doi: 10.1371/journal.pone.0026369 (PMC3192797; doi:10.1371/journal.pone.0026369)
Supplement: Table S2 — Genes identified by GSEA as contributing significantly to the assessment of enrichment for the PBX1 binding motif in Type II-induced genes. Gene set enrichment analysis (GSEA) was used to find candidate transcription factors induced upon infection; the reference gene set used was c3.tft.v3.0 from the Molecular Signatures Database (comprised of gene sets predicted on the basis of a common cis-regulatory motif conserved in the human, mouse, rat, and dog genomes). A PBX1 motif was identified as significantly enriched (at the FDR<0.25 level) in genes highly expressed during Type II infection. The subset of genes identified as contributing most significantly to this enrichment are listed in the table by gene symbol, along with their rank in gene list (i.e. the position of the gene in the ranked list of genes), rank metric score (correlated to the fold change of gene expression in Type III vs. Type II infection), and running ES (enrichment score). The running ES here indicates the degree to which the reference gene set is overrepresented at the top or bottom of the ranked list of genes differentially expressed in Type II versus III infection. A negative ES indicates gene set enrichment at the bottom of the list, i.e. among genes more highly expressed during Type II infection. (DOCX) [file pone.0026369.s002.docx]

**Table S2.**

| GENE SYMBOL | RANK IN GENE LIST | RANK METRIC SCORE | RUNNING ES |
| --- | --- | --- | --- |
| EML1 | 11458 | -0.19159174 | -0.5895484 |
| SLC43A3 | 11517 | -0.198713303 | -0.5829971 |
| BCL9 | 11550 | -0.202373981 | -0.5744073 |
| CCNG1 | 11732 | -0.2243433 | -0.5751957 |
| USP47 | 11802 | -0.234480858 | -0.56750494 |
| ZIC1 | 11950 | -0.253847122 | -0.56430155 |
| TRIB2 | 12515 | -0.338944435 | -0.586078 |
| ZHX2 | 12612 | -0.355079889 | -0.57383025 |
| SLITRK2 | 12665 | -0.364488363 | -0.5579602 |
| CNOT7 | 12736 | -0.377420902 | -0.54267174 |
| RPA3 | 13408 | -0.544630051 | -0.56099457 |
| AKAP12 | 13587 | -0.619073153 | -0.54039377 |
| DUSP10 | 13678 | -0.669480801 | -0.5108537 |
| TLE4 | 13795 | -0.752474308 | -0.47870332 |
| LIF | 13848 | -0.791224003 | -0.43993956 |
| PPM1D | 13857 | -0.800467491 | -0.3975624 |
| RNF128 | 13885 | -0.821559906 | -0.35539988 |
| RHOB | 13909 | -0.864931107 | -0.31062713 |
| CYB5R2 | 13999 | -0.991136074 | -0.26375988 |
| RARB | 14011 | -1.011142254 | -0.2102929 |
| PITX2 | 14056 | -1.169525146 | -0.150667 |
| HES1 | 14066 | -1.21036911 | -0.08637007 |
| MMP1 | 14157 | -1.782928944 | 0.002904899 |
